# The Working Hours of the Private Nurse

**Published:** 1919-05-24

**Authors:** Geraldine Bremner

**Affiliations:** 22 Langham Street, Portland Place, W. 1


### THE WORKING HOURS OF THE PRIVATE NURSE.

*To the Editor of* The Hospital.

Sir,—At last someone has been sympathetic and brave enough to raise the question for discussion of the hours on duty of a private nurse—I refer to Miss Sheldon's letter in your issue of May 17. Up to this time, although there has been much written and much discussion about nurses' hours on duty, etc., the private nurses' interests in these respects have been entirely ignored—so much so that a visitor from Mars might imagine no such branch of nursing existed. This is surprising, seeing that the branch in question embraces such numbers of the profession. It also emphasises the importance of direct representation by private and district nurses on the Council of the College of Nursing. There is no doubt that it is a question that seems full of difficulties, and at times impossible of solution. I think it can only be solved by a bold move—viz., legislation for the hours of *all* nurses, no matter whether they be hospital, private, or district nurses. When it is thoroughly understood that a nurse is only *expected* to be on duty for a certain number of hours—say, eight or ten—many other difficulties will disappear. In private work much could be left to the nurse herself. No nurse worthy of the name would allow her patient to be left unattended during a dangerous or acute stage of illness, but after that period was over it would be quite possible for her to work her stipulated hours only, and many patients and doctors would be glad to help her to do this, knowing that she had willingly given extra time and care to her patient at a time when such close attention was really necessary. In many minor cases of illness the attention of a nurse for eight or ten hours is quite sufficient. I would suggest that all private nursing institutions should get their nurses under an eight or ten hour Bill, and for the rest trust the nurse to do her best where difficulties arise. Action of this sort would tend to bring out the spirit of the ministering angel, and would retain in as well as attract to this branch of the service some of the best workers.

Yours faithfully,
Geraldine Bremner.
22 Langham Street, Portland Place, W. 1, May 19, 1919.

